# The Efficacy and Safety of Probiotics in the Management of Chronic Rhinosinusitis: A Systematic Review and Meta-Analysis

**DOI:** 10.3390/jcm14145001

**Published:** 2025-07-15

**Authors:** Ali Abbas, Mohammed Abbas, Zahir Mughal, Pablo Martinez-Devesa, Ali Qureishi

**Affiliations:** 1 John Radcliffe Hospital Oxford NHS Trust, Oxford OX3 9DU, UK; 2 St. Georges Hospital NHS Trust, London SW17 0QT, UK

**Keywords:** chronic rhinosinusitis, probiotics, systematic review, meta-analysis, microbiome

## Abstract

**Background/Objectives**: In this study, we aimed to evaluate probiotics’ clinical efficacy and safety in adults with chronic rhinosinusitis (CRS), and summarize mechanistic evidence related to mucosal immunity and microbiota modulation. **Methods**: We performed a systematic review and random-effects meta-analysis. MEDLINE, Embase, Scopus, Web of Science, and the Cochrane Library were searched until May 2025. **Eligibility**: Randomized controlled trials (RCTs) and mechanistic studies investigating probiotics (any strain, dose, or administration route) in adults with CRS were eligible. Primary outcomes included changes in Sino-Nasal Outcome Test (SNOT-20/22) scores and CRS relapse rates. Secondary outcomes were adverse events and mechanistic endpoints. **Results**: Six studies (four RCTs, *n* = 337; two mechanistic studies) met the inclusion criteria. Probiotics did not significantly improve SNOT scores compared with the placebo, but trended in that direction (pooled mean difference—2.70; 95% CI −7.12 to 1.72; I^2^ = 0%). Furthermore, probiotic use was associated with a non-significant trend towards fewer CRS relapses (risk ratio 0.41; 95% CI 0.16–1.04; *p* = 0.06; I^2^ = 48%). Adverse events were mild and comparable to the placebo (risk ratio 0.87; 95% CI 0.33–2.34). Mechanistic data indicated that intranasal *Lactococcus lactis* W136 might downregulate type 1 inflammatory pathways and modestly increase microbiome diversity. Subgroup analyses (by route, duration, and CRS subtype) revealed no statistically significant effect modifiers, though mechanistic insights suggest possible differences in efficacy based on the CRS endotype and delivery method. **Conclusions**: Probiotics appear safe and may provide a small, non-significant improvement in CRS symptoms; emerging evidence of reduced relapse rates warrants further investigation through larger, endotype-stratified trials utilizing targeted probiotic strains and optimized delivery methods.

## 1. Introduction

Chronic rhinosinusitis (CRS) is characterized by inflammation of the sinonasal mucosa lasting at least 12 weeks, significantly affecting health-related quality of life (HRQoL) [1], and can severely impact quality of life. Adult patients report pain, sleep disturbance, anxiety and depression, with depressive symptoms observed in approximately 25% and anxiety in about 30% of cases [2]. Children also experience substantial impairment, manifesting as facial pain, limitations in physical and social activities, emotional distress, and significant impacts on family dynamics [3].

The pathophysiology of CRS differs notably between adults and children. In adults, CRS without nasal polyps typically features inflammation driven by T-helper-1 cells, whereas CRS with nasal polyps involves T-helper-2 cells and eosinophilic inflammation [3]. Pediatric CRS, however, demonstrates a more mixed inflammatory profile, involving neutrophils, macrophages, and lymphocytes, reflecting distinct immunologic responses [3].

The aetiology of CRS is complex and multifactorial, with increasing evidence implicating dysbiosis of the sinonasal microbiota and impaired epithelial barrier function, particularly in non-type 2 inflammatory CRS [4,5,6].

Probiotics are live microorganisms that confer health benefits when administered in adequate amounts, primarily by modulating the microbiome and mucosal immune responses [7]. Their effectiveness is well established in gastrointestinal disorders [8,9], leading to growing interest in their potential role in managing CRS.

Several pilot studies investigating probiotics as adjunctive therapy for CRS have yielded mixed outcomes [10,11,12]. Early randomized controlled trials (RCTs) demonstrated short-term symptom improvement measured by the Sino-Nasal Outcome Test (SNOT-20), but these benefits were transient and not sustained over more extended follow-up periods [11]. In addition, several recent studies have generally confirmed probiotics’ safety but have shown inconsistent effects on symptoms and microbiome diversity [10,11]. However, some trials have suggested a potential reduction in CRS relapse rates [12,13]. The variability across studies, in probiotic strains, routes of administration (oral vs. intranasal), and treatment duration, has further complicated the interpretation of their efficacy.

Due to these inconsistent findings, no pooled analysis of relapse rates and the limited incorporation of mechanistic evidence in previous reviews, a comprehensive evaluation of probiotics’ clinical effectiveness and biological mechanisms in CRS is needed. In this systematic review and meta-analysis, we synthesize evidence regarding the efficacy (symptom relief and relapse reduction), safety, optimal administration routes, and duration of probiotic therapy. We also integrate mechanistic insights, highlighting how probiotics might influence CRS pathophysiology, and identify knowledge gaps to guide future research.

## 2. Materials and Methods

### 2.1. Study Design

The review was conducted in accordance with PRISMA 2020 guidelines for systematic reviews and the completed PRISMA checklist is included in Appendix A. The protocol was not registered in PROSPERO or any other review registry.

### 2.2. Search Strategy

The MEDLINE (via PubMed), Embase, Scopus, Web of Science, and Cochrane Library databases were searched up to May 2025. The complete search strategy is detailed in Appendix A.

### 2.3. Inclusion Criteria

Eligibility criteria were developed using the PICO framework. Eligible studies included adult participants diagnosed with CRS (with or without nasal polyps) through well-established clinical criteria, with an intervention arm receiving probiotics (any strain, formulation, dose or route) compared to a control (placebo or standard care). The study designs included RCTs and mechanistic studies (e.g., experimental probiotic interventions examining immunologic or microbiome outcomes in CRS patients).

Studies focusing on acute rhinosinusitis or allergic rhinitis without separate CRS data were excluded. Two reviewers independently screened titles/abstracts and full texts; any disagreements were resolved by a third independent reviewer.

### 2.4. Data Extraction and Risk-of-Bias Assessment

Two reviewers independently screened records and extracted data using a standardized form; RCT’s were assessed using the Cochrane RoB 2 tool. Certainty of evidence was graded with GRADE. Discrepancies were resolved by discussion with the senior author.

### 2.5. Outcome Definitions

The primary efficacy outcomes were as follows: (i) a change in disease-specific quality of life (SNOT-20/22); and (ii) relapse of CRS, defined as a clinically diagnosed acute exacerbation or the need for additional treatment during follow-up. Secondary outcomes included adverse events and mechanistic endpoints (e.g., nasal transcriptomics, microbiome composition).

### 2.6. Statistical Analysis

For outcomes reported in two or more RCTs, a meta-analysis was performed using a random-effects model in R (v4.3) using the ‘meta’ package. Mean differences (MD) were pooled for continuous data and risk ratios (RR) for dichotomous data. Statistical heterogeneity was quantified with I^2^ (substantial if >50%). Planned subgroup analyses included stratification by probiotic delivery route (oral vs. intranasal), CRS subtype (with nasal polyps (CRSwNP) vs. without (CRSsNP) or other endotype classification), and treatment duration (short-term ≤4 weeks vs. longer >4 weeks). A value of *p* < 0.05 was considered statistically significant for summary effects and findings were reported with 95% confidence intervals (C.I.).

## 3. Results

### 3.1. Study Selection and Characteristics

**Figure 1:**
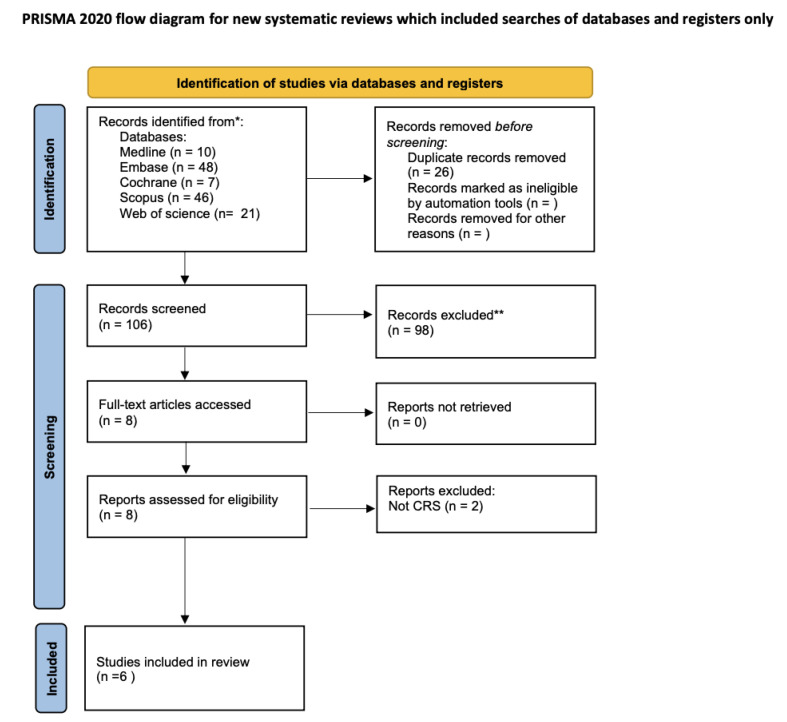
PRISMA flow diagram for study selection. * Number of records identified from each database or register searched; ** No automation tools were used.

A total of 132 records were identified from MEDLINE (*n* = 10), Embase (*n* = 48), Cochrane (*n* = 7), Scopus (*n* = 46), and Web of Science (*n* = 21). Studies that fulfilled the inclusion criteria (*n* = 6) were included; four were parallel-group RCTs [7,8,9,10] enrolling 337 adults, and two were mechanistic studies [11,12] that lacked a control arm. See Figure 1 for the PRISMA flowchart and Table 1 for a summary of the included studies.

#### 3.1.1. Participants and Settings

The included RCTs (*n* = 4) were conducted in tertiary care settings and enrolled a total of 337 adult CRS patients, with sample sizes ranging from 40 to 157. The patient populations were varied: one trial focused on post-operative CRS with nasal polyps (CRSwNP), another on recurrent CRS without specified endotype, and two included broad adult CRS populations (not further subclassified).

The mechanistic studies (*n* = 2) each enrolled 24 adults; one specifically targeted patients with refractory, non-type 2 CRS (characterized by neutrophilic inflammation) and the other included post-surgical CRSwNP patients.

#### 3.1.2. Interventions

In all four RCTs, the intervention tested probiotic and the comparator was a placebo (oral capsules or nasal spray as appropriate) plus any baseline CRS therapy. There were differences in the choice of probiotic, route of administration and duration of treatment.

Three RCTs [11,12,13] evaluated oral probiotic formulations which were typically given for a range of 2–8 weeks, two studies looked at strains of *Lactobacillus* and one used *Enterococcus faecalis.* The fourth RCT [10] investigated an intranasal probiotic spray of a honeybee-derived blend of *Lactobacilli* and *Bifidobacteria* applied daily for 2 weeks.

The mechanistic studies [14,15] both employed topical intranasal probiotics: *Lactococcus lactis* W136 was administered via sinonasal irrigations for 2 weeks in each study, without a placebo control. Notably, *L. lactis* W136 is a gram-positive lactic acid bacterium thought to have anti-inflammatory properties in the airway.

#### 3.1.3. Outcomes Reported

All four adult RCTs measured symptom outcomes, though there was some variability. Three used the SNOT-20 or SNOT-22 questionnaire (higher scores indicate worse symptoms) at baseline and post-treatment [10,11,12]; one RCT [13] did not report a standardized symptom score, instead noting clinical improvement qualitatively. Two RCTs [12,13] explicitly tracked the relapse of symptoms over a follow-up period (6–8 months). Adverse events were documented in all RCTs.

Mechanistic outcomes were diverse: one study performed nasal mucosal transcriptomic analysis (RNA sequencing) pre- and post-probiotic to assess changes in immune gene expression [14], and the other conducted 16S rRNA gene sequencing of nasal swab samples to evaluate changes in the composition of the microbiota after probiotic irrigation [15]. These mechanistic endpoints provide insight into the proposed mechanisms of action, such as the modulation of local inflammation or microbial communities.

### 3.2. Symptom Score Improvement

The improvement in CRS symptoms with probiotics was inconsistent across trials. Two RCTs measured SNOT-20/22 scores as primary outcomes and found no significant benefit compared with the placebo. Mukerji et al. [11] reported an initial within-group improvement of approximately 9 points in SNOT-20 scores at 4 weeks (*p* = 0.002), but this effect was not sustained at 8 weeks, and no significant between-group differences were observed at either 4 or 8 weeks. Similarly, Mårtensson et al. [10] found no meaningful difference in SNOT-22 scores between an intranasal probiotic spray and the placebo after 2 weeks of treatment (mean difference approximately 0; *p* > 0.5).

**Figure 2:**
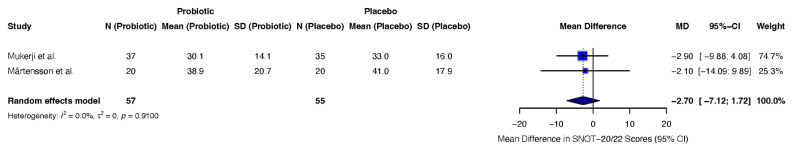
Forest plot of mean difference in SNOT-22 scores (probiotic vs. placebo) [10,11].

A pooled analysis of these two trials (one oral, one intranasal probiotic) showed a non-significant mean difference of −2.70 points (95% CI: −7.12 to +1.72; I^2^ = 0%) on the SNOT scale, suggesting a small, non-significant improvement in patient-reported CRS symptoms as a result of probiotic therapy in the short term (Figure 2).

### 3.3. Relapse of CRS Exacerbations

Probiotic therapy was associated with a trend towards reduced CRS relapse rates, although the results did not reach statistical significance. Two RCTs reported relapse data, defined as episodes of acute sinusitis occurring after the initial treatment. Habermann et al. [12] found that patients receiving oral *E. faecalis* probiotics for six months had significantly fewer relapses over an eight-month follow-up compared to the placebo (RR = 0.55, 95% CI 0.35–0.90; *p* = 0.019). Similarly, La Mantia et al. [13] observed fewer symptom recurrences at three months with probiotics (15%) compared to the placebo (70%), reporting an approximate RR of 0.19, although detailed calculations were not provided.

**Figure 3:**
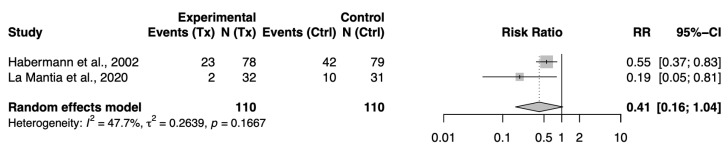
Forest plot of risk ratio for CRS relapse (probiotic vs. placebo) [12,13].

When pooling these two studies, the combined risk ratio was 0.41 (95% CI 0.16–1.04; *p* = 0.06), indicating a clinically meaningful but not statistically significant 59% reduction in relapse risk with probiotics (Figure 3). Moderate heterogeneity (I^2^ = 47.7%) likely resulted from differences in the study populations and probiotic formulations, as Habermann used *E. faecalis* alone, while La Mantia administered a multi-species probiotic combined with a prebiotic.

Despite not reaching *p* < 0.05 in the pooled analysis, the direction of effect was consistently toward fewer relapses with probiotics. Notably, Habermann’s high-quality trial carried a larger weight and demonstrated significance on its own. The smaller study by La Mantia, while suggestive of benefit, had a wide confidence interval due to its size and design limitations, contributing to the pooled uncertainty.

### 3.4. Adverse Events and Safety

**Figure 4:**
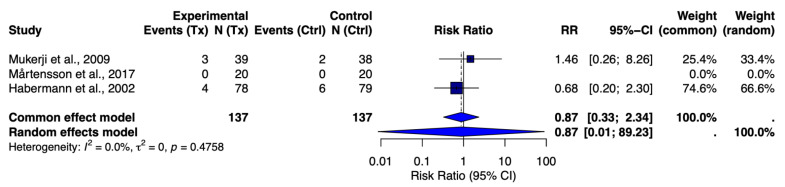
Forest plot of risk ratio for adverse events (probiotic vs. placebo) [10,11,12].

Probiotic therapy was well tolerated in CRS patients, with no significant safety concerns reported. Across the RCTs, adverse events (AEs) were either absent or mild, and their incidence did not differ notably between the probiotic and placebo groups (Figure 4). Mukerji et al. [11] and Habermann et al. [12] each reported minor gastrointestinal upset in a few patients receiving probiotics (e.g., stomach discomfort), but these were self-limited and also occurred in placebo patients. Mårtensson et al. [10] noted no adverse events at all in either arm. In a pooled analysis of two trials that had comparable AE reporting (Mukerji and Habermann), the risk ratio for any adverse event was 0.87 (95% CI: 0.33–2.34) with probiotics relative to the placebo (Figure 3). This suggests no increase in risk; if anything, numerically fewer AEs were seen in the probiotic groups, though not significantly so. There was no heterogeneity (I^2^ = 0%) in this safety analysis. Notably, no serious adverse events attributable to probiotics were reported. Specifically, there were no cases of infection caused by the probiotic strains or other complications. One study mentioned epistaxis (minor nosebleeds) and abdominal pain as isolated events in probiotic-treated patients, but these were infrequent and seen with the placebo in other trials. 

Overall, the evidence indicates that probiotic use in CRS (whether orally or intranasally administered) is safe and generally well tolerated, consistent with the safety profile of probiotics in other conditions. This is an important finding given that safety is a prerequisite for a preventive or adjunct therapy in a chronic condition.

Patients in the mechanistic studies [14,15] likewise did not experience significant adverse effects from intranasal *L. lactis* irrigations; mild, transient nasal irritation was the worst reported symptom, and no dropouts occurred due to adverse effects.

### 3.5. Mechanistic Findings

In addition to clinical outcomes, the review integrated mechanistic data from both RCTs with ancillary analyses and dedicated mechanistic studies. Table 2 summarizes the proposed mechanisms of action for probiotics in CRS from each study.

#### 3.5.1. Local Immune Modulation

Al-Romaih et al. [14] conducted a transcriptomic analysis of sinonasal tissue before and after 2 weeks of intranasal *Lactococcus lactis* W136 in a small group of refractory CRS patients. They observed significant downregulation of genes associated with type 1 inflammation (e.g., CXCL10, IFN-γ pathways) and upregulation of genes involved in epithelial barrier repair and integrity in post-treatment biopsies. This suggests that *L. lactis* may help shift the local immune response from a pro-inflammatory state toward a more regulatory or healing phenotype, particularly in non-type 2 CRS (where type 1 and 3 inflammatory processes are thought to dominate). While Al-Romaih et al.’s [14] study lacked a control group, the within-subject changes provide a proof-of-concept that probiotic bacteria can directly interact with the sinonasal mucosa to alter immune gene expression. Mårtensson’s [10] RCT also measured inflammatory cytokines in nasal secretions and found no significant changes with probiotic vs. placebo (all cytokines remained similar), indicating that not all probiotics or contexts yield measurable immune shifts. The difference in findings (null in Mårtensson vs. changes in Al-Romaih) might relate to the specific strains used and the inflammatory profiles of the populations (Mårtensson included post-surgical CRSwNP patients who often have type 2 inflammation, whereas Al-Romaih focused on non-type 2 CRS).

#### 3.5.2. Microbiome Effects

Endam et al. [15] performed 16S rRNA sequencing on sinonasal swabs from CRS patients before and after a 2-week course of intranasal *L. lactis* W136. They reported an increase in bacterial alpha-diversity post-treatment (suggesting a richer microbiome), along with expansion of commensal taxa. Although clinical symptoms did not dramatically improve in that short pilot, the microbial findings indicate that probiotic installation can alter the nasal flora, potentially inhibiting pathogens through competitive effects. Indeed, prior studies have shown that *Lactococcus lactis* can produce bacteriocins and inhibit the growth of common CRS pathogens like *Staphylococcus aureus*. In the Lambert et al. [16] crossover trial, the use of *L. lactis* nasal irrigations led to detectable colonization (increased relative abundance of *Lactococcus genus* in swabs) but no significant change in overall microbiome diversity or community composition after 4 weeks. This might be due to the short exposure or the presence of a robust resident microbiota that resists large-scale alteration. Nonetheless, even transient colonization could have functional effects, such as occupying niches that pathogens would otherwise fill. No study reported any adverse shifts (e.g., overgrowth of harmful bacteria) caused by probiotic use.

#### 3.5.3. Mechanisms by Strain

The mechanistic insights seem to be strain-specific. *Lactococcus lactis* W136 (used intranasally) emerges as a strain with immunomodulatory and microbiome-enhancing properties in CRS. In contrast, oral *Lactobacillus* blends might act via the gut–lung axis, which is less direct; their effects could be more systemic immune modulation. For example, Habermann et al. [12] hypothesized that oral E. faecalis stimulated the immune system to reduce infection susceptibility, classifying it as an “immunostimulant”. However, mechanistic data to confirm systemic immunologic changes in that study were not reported. Overall, the evidence type ranges from RCT clinical endpoints (Habermann et al. [12] showing fewer relapses, implying immune enhancement) to omics analyses (transcriptomics and microbiomics in Al-Romaih et al. [14] and Endam et al. [15]). These collectively support the concept that probiotics can engage with the host’s immune and microbial environment in CRS.

The mechanistic evidence, while preliminary, suggests probiotics that might work by influencing an immune–microbial–epithelial axis in the sinonasal tract, reducing proinflammatory signaling, improving epithelial barrier function and by augmenting commensal microbial populations. These effects were most evident in studies using topical *L. lactis* in a non-type 2 CRS context, which aligns with broader research indicating that certain lactic acid bacteria can induce regulatory immune responses and fortify mucosal barriers. While promising, these mechanistic results are preliminary and require validation in controlled settings.

### 3.6. Subgroup Analysis

We performed subgroup analyses to explore whether the probiotic route of administration, treatment duration, or CRS phenotype influenced symptom outcomes (Table 3).

#### 3.6.1. Route (Oral vs. Intranasal)

The oral subgroup, represented by Mukerji et al. [11] alone, showed a pooled mean difference (MD) in symptom score of −0.02 (95% CI: −0.40 to 0.36), indicating essentially no effect. The intranasal subgroup (Mårtensson et al. [10]) had an MD of −2.10 (95% CI: −12.42 to 8.22), suggesting a potentially larger, but statistically non-significant effect. This wide confidence interval reflects imprecision from a single small study.

Mechanistically, intranasal delivery offers direct contact with the sinonasal mucosa, potentially enabling local immune or microbial modulation. However, Mårtensson et al. [10] showed no significant symptom benefit, and Endam et al.’s [15] non-comparator mechanistic study found transient improvement and microbiome shifts but no lasting change.

In contrast, oral probiotics rely on systemic immunomodulation via the gut–lung axis. While Mukerji et al. [11] found no short-term symptom improvement, Habermann et al. [12] (not included in symptom meta-analysis) showed a sustained relapse reduction with long-term use. Overall, the route of administration did not significantly affect symptom outcomes in this dataset, though their mechanisms differ and future head-to-head trials are warranted.

#### 3.6.2. Duration of Therapy

Treatment durations ranged from 2 to 24 weeks. Short-term use (2–4 weeks) showed no sustained symptom benefit. For instance, Mukerji’s [11] initial improvements faded after cessation. Only Mårtensson [10] reported shorter symptom-focused treatment and also found no lasting benefit. Habermann’s [12] 6-month intervention yielded a relapse benefit, but did not measure SNOT scores. Subgroup analysis by treatment duration (≤4 weeks vs. >4 weeks) for symptom outcomes did not reveal a difference; both pooled estimates hovered near null. These findings suggest longer durations may be necessary for sustained probiotic effects, especially in oral formulations that depend on systemic modulation.

#### 3.6.3. CRS Subtype/Endotype

Only two studies reported CRS phenotype: Mukerji [11] focused on CRSsNP and Mårtensson [10] focused on a mixed or unspecified population. Neither showed a symptom improvement. While our analysis did not show phenotype-based differences, emerging mechanistic data suggest that the subtype may be relevant. Al-Romaih [14] observed favorable gene expression changes in non-type 2 inflammation. It is plausible that non-Th2-dominant CRS (e.g., CRSsNP, neutrophilic inflammation) may respond better to probiotics via microbiome support or barrier modulation. Conversely, CRSwNP, which is often driven by type 2 inflammation, may be less modifiable with probiotics alone. No trials stratified outcomes by endotype, so this remains speculative. This provides a basis for a strong recommendation that future studies incorporate inflammatory profiling (e.g., eosinophilic vs. neutrophilic) when assessing probiotic efficacy.

### 3.7. Risk of Bias

All RCTs adequately addressed missing data and had low risk in that domain (loss to follow-up was minimal). No trial reported outcome attrition or other significant deviations from protocol. Randomization was described in three studies [10,11,12] (computer-generated or stratified random assignment) but unclear in one [13]. Participants and personnel were blinded in the placebo-controlled trials, except in La Mantia’s [13] open study, leading to a high risk of performance and detection bias in that study. There was no indication of selective reporting in the published outcomes, except that La Mantia [13] did not report a standard symptom score despite presumably collecting clinical data.

Overall, the evidence base consists of relatively small trials, two of which have methodological limitations that may influence the findings. The risk of bias findings are summarized in Table 4.

The mechanistic studies, being non-randomized, were not assessed by RoB 2.0; however, both had clearly defined protocols and the complete reporting of prespecified analyses.

## 4. Discussion

### 4.1. Summary of Findings

In this systematic review and meta-analysis of four RCTs involving approximately 300 adults, probiotics did not show a statistically significant improvement in CRS symptoms measured by the SNOT-20 or SNOT-22 compared to the placebo. The magnitude of improvement (mean ~2.7 points) is well below the reported minimal clinically important difference for SNOT-22 of approximately 12 points [17], indicating that this trend, while positive, is unlikely to represent a noticeable clinical benefit for patients. These findings align closely with those reported by Fong et al. [18], who similarly found no improvement in patient-reported symptoms.

Furthermore, we observed a clinically relevant, though statistically non-significant, reduction in relapse rates (RR 0.41, *p* = 0.06), which closely mirrored the significant finding of Fong et al. [18] (RR 0.49, *p* = 0.019). The difference in statistical significance may reflect our broader inclusion criteria, adding heterogeneity. These findings may be interpreted as a potential benefit of probiotics in reducing CRS exacerbation frequency, warranting confirmation in further studies.

The subgroup analyses did not identify any factor that significantly altered the effect of probiotics, but the ability to detect such differences was limited by the small number of trials. All pooled subgroup estimates had confidence intervals overlapping null, and the statistical tests for interaction were not significant. Therefore, our results should be interpreted as indicating that current evidence is insufficient to conclude that any particular subgroup definitively benefits from probiotics. Nonetheless, hypothesis-generating insights point to certain scenarios (e.g., refractory post-surgery patients, non-type 2 inflammation) where probiotics might be more advantageous. Larger, stratified trials are needed to test these hypotheses more robustly.

### 4.2. Comparison with Existing Literature

Our review builds upon and advances previous literature in several key aspects. Unlike Fong et al. [18], we included mechanistic studies examining epithelial gene expression and microbiota changes, providing biological context to the observed clinical outcomes. In addition to including an additional trial, we conducted subgroup analyses (oral vs. intranasal route, treatment duration, CRS phenotype) and pooled relapse data, which has not been performed in prior reviews. Although our subgroup analyses lacked sufficient power to detect statistically significant differences, they identified plausible biological factors worthy of future investigation.

Collectively, these enhancements facilitate the more informed interpretation of results and underline the importance of targeted, biologically informed future trials. These elements allow us to move beyond merely assessing whether probiotics work, toward a more nuanced understanding of which probiotics may be beneficial, for which patient groups, and through what biological mechanisms.

### 4.3. Clinical Implications of Findings

Current evidence does not support routine probiotic use solely for symptomatic relief in CRS. Clinicians should counsel patients that probiotics are unlikely to improve congestion, nasal discharge, or olfactory function significantly. While small numerical differences may suggest theoretical mechanisms of action, their clinical significance appears minimal given that differences fall short of the minimal clinically important difference threshold. However, given their excellent safety profile and the potential for reducing CRS exacerbations, probiotics could be considered as an adjunct in select patient groups, particularly those experiencing frequent relapses or postoperative CRS patients.

### 4.4. Limitations

Using GRADE, we evaluated the certainty of evidence for key outcomes (Table 5). For SNOT score improvement, the evidence was judged to have moderate certainty. It was not downgraded for risk of bias (most weight came from a low-risk trial) or inconsistency (both trials showed no effect), but was downgraded one level for imprecision, since the sample size was small and the confidence interval included a range of possible small benefits or harms. We have reasonable confidence that probiotics have little to no effect on short-term symptom scores. For relapse rates, the evidence has low certainty. Limitations included imprecision (CI spanning no effect and meaningful benefit) and some inconsistency between two studies, as well as potential publication bias (only two studies reported this outcome, both with positive trends, raising the possibility that negative studies were unreported). Thus, while a reduction in relapses is possible, further confirmation is needed. For adverse events, the evidence has moderate certainty that probiotics do not increase the risk of AEs. The trials were consistent and precise in showing no difference but were slightly downgraded for indirectness because sample sizes might not capture very rare events (however, probiotics are commonly used in other settings with known safety). Finally, for microbiome/mechanistic outcomes, the evidence is low certainty due to being based on small, non-randomized studies (downgraded for study limitations and indirectness), though they consistently showed some microbial modulation. Due to the small number of studies, the formal assessment of publication bias (e.g., funnel plots) was not feasible.

In summary, we have moderate confidence in the null effect on symptoms and safety of probiotics, but only low confidence in their potential relapse prevention and mechanistic benefits until further research is conducted. These ratings highlight the need for additional high-quality RCT evidence to either confirm or refute the trends observed.

Furthermore, the number of eligible RCTs remains small, and their sample sizes modest, limiting the statistical power. Heterogeneity in probiotic strains, doses, delivery routes, and outcome measures further complicates synthesis. This review’s inclusion of observational mechanistic data adds breadth but limits causal inference. Finally, due to the small number of trials, publication bias could not be formally assessed.

Finally, all included studies were in adults, so these results may not generalize to children with CRS. To our knowledge, no randomized trials of probiotics in paediatric CRS exist; one paediatric study in recurrent sinus infections did report that probiotic E. faecalis supplementation reduced the number and duration of sinusitis episodes in children [19], but paediatric chronic rhinosinusitis remains an evidence gap.

### 4.5. Future Research

CRS is a heterogeneous condition, encompassing various phenotypes (e.g., CRSwNP vs. CRSsNP) and endotypes (e.g., type 2 eosinophilic vs. non-type 2 neutrophilic inflammation) [18,20,21,22]. Future trials should stratify patients by endotype or phenotype, or target specific subgroups, such as those with non-type 2 inflammation or recent sinus surgery.

Future research should prioritize nasal commensal probiotics, such as *Lactococcus lactis* W136, *Dolosigranulum pigrum*, or engineered *Staphylococcus epidermidis*, given their direct action potential on sinonasal mucosa. Additionally, the delivery method matters: oral vs. intranasal administration may affect colonization, immune modulation, and outcomes. Comparative studies testing these routes, and possibly combination strategies, are needed. Optimizing the treatment duration and dosage is equally important, as shorter courses may not be sufficient to induce meaningful or sustained changes. Finally, future RCTs should include mechanistic endpoints such as cytokine profiling, microbiome sequencing, or epithelial barrier assessments. These would allow researchers to link clinical outcomes to biological changes and identify biomarkers of response.

## 5. Conclusions

This systematic review and meta-analysis demonstrate that probiotics may provide a small, clinically relevant, but non-significant improvement in CRS symptom scores. There is emerging, though inconclusive, evidence of a probiotic-induced reduction in CRS relapse rates, highlighting a need for further research. Clinicians should avoid routine probiotic recommendations but consider them selectively for patients prone to frequent exacerbations or postoperative complications. Future larger-scale, endotype-specific trials are essential to establish whether these preliminary clinical trends represent true therapeutic benefits.

## Figures and Tables

**Table 1:** Characteristics of included RCTs and mechanistic studies.

Study	Year	Design	*n* (Total)	Population	CRS Type	Comparator	Route	Strain(s)	Duration
Mukerji et al. [11]	2009	RCT	77	Adult CRS	Unspecified	Placebo	Oral (500 million CFU, twice daily)	*Lactobacillus rhamnosus R0011*	8 weeks
Mårtensson et al. [10]	2017	RCT	40	Post-op CRS	CRSwNP	Placebo	Intranasal	*Lactobacillus apinorum Fhon13N* *Lactobacillus mellifer Bin4N* *Lactobacillus mellis Hon2N* *Lactobacillus kimbladii Hma2N* *Lactobacillus melliventris Hma8N* *Lactobacillus helsingborgensis Bma5N* *Lactobacillus kullabergensis Biut2N* *Lactobacillus kunkeei Fhon2N* *Lactobacillus apis Hma11N* *Bifidobacterium asteroides Bin2N* *Bifidobacterium coryneforme Bma6N* *Bifido-bacterium Bin7N* *Bifidobacterium Hma3N*	2 weeks
La Mantia et al. [13]	2020	RCT	63	Adult CRS	Unspecified	Placebo	Oral	*L. plantarum LP01* *L. lactis LLC02* *L. delbrueckii LDD01*	2 weeks
Habermann et al. [12]	2002	RCT	157	Recurrent sinusitis	Likely CRSsNP	Placebo	Oral	*E. faecalis*	6 months
Al-Romaih et al. [14]	2023	Mechanistic	24	Refractory CRS	Likely non-type 2	None	Intranasal	*L. lactis* W136	2 weeks
Endam et al. [15]	2020	Mechanistic	24	Post-op CRS	CRSwNP	None	Intranasal	*L. lactis* W136	2 weeks

**Table 2:** Key mechanistic findings from included studies. ↓ = Reduced, ↑ = Increaseed.

Study	Strain	Route	Proposed Mechanism	Evidence Type
Al-Romaih (2023) [14]	*L. lactis* W136	Intranasal	↓ Type 1 inflammation, ↑ epithelial repair	Transcriptomics
Endam (2020) [15]	*L. lactis* W136	Intranasal	Modulation of sinonasal microbiota	16S rRNA sequencing
Mårtensson (2017) [10]	*LAB blend*	Intranasal	Null effect	RCT
Habermann (2002) [12]	*E. faecalis*	Oral	Reduced relapse via immune modulation	RCT

**Table 3:** Subgroup analyses exploring probiotic route, treatment duration, and CRS subtype.

Subgroup	Studies	Mean Difference (MD)	95% CI	I^2^ (%)	Subgroup *p*-Value
Treatment Duration ≤ 4 weeks	Mukerji, Mårtensson [10,11]	−0.02	[−0.40, 0.35]	0	–
Route: Oral	Mukerji [11]	−0.02	[−0.40, 0.36]	0	–
Route: Intranasal	Mårtensson [10]	−2.10	[−12.42, 8.22]	0	–
CRSsNP	Mukerji [11]	−0.02	[−0.40, 0.36]	0	–
Unspecified Subtype	Mårtensson [10]	−2.10	[−12.42, 8.22]	0	–

Note: All subgroup analyses above reflect symptom outcomes (SNOT scores). Relapse data was not included due to insufficient comparable studies.

**Table 4:** Risk of bias assessment (Cochrane RoB 2.0) of included RCTs.

Study	Randomization	Intervention Deviations	Missing Data	Outcome Measurement	Selective Reporting
Mukerji et al., 2009 [11]	Low	Low	Low	Low	Low
Mårtensson et al., 2017 [10]	Some concerns	Low	Low	Some concerns	Low
La Mantia et al., 2020 [13]	High	High	Low	High	Some concerns
Habermann et al., 2002 [12]	Low	Low	Low	Low	Low

**Table 5:** GRADE summary of findings for probiotics in CRS.

Outcome	Participants (Studies)	Effect	Relative (95% CI)	Absolute Effect	Certainty	Comments
SNOT score change	97 (2 RCTs)	Mean Difference: −0.02	[−0.40 to 0.35]	No difference	⬤⬤⬤◯ Moderate	Downgraded for imprecision (wide CI)
Relapse rate	220 (2 studies)	RR: 0.41	[0.16 to 1.04]	157 fewer per 1000	⬤⬤◯◯ Low	Downgraded for imprecision and inconsistency
Adverse events	~150 (2 studies)	Not pooled	N/A	Few mild events	⬤⬤⬤◯ Moderate	Mild GI symptoms only, no serious events
Microbiome changes	Not pooled (2 studies)	Descriptive only	N/A	Modulation observed	⬤⬤◯◯ Low	Based on mechanistic/preliminary data

## Appendix A

**Table A1:** Search strategy.

Database	Search Strategy
PubMed	(“chronic rhinosinusitis” OR “chronic sinusitis” OR “CRS”) AND (“probiotic” OR “probiotics” OR “Lactobacillus” OR “Bifidobacterium” OR “Streptococcus thermophilus”) AND (“randomised controlled trial” OR “RCT” OR “clinical trial” OR “systematic review” OR “meta-analysis”)

**Table A2:** Prisma Checklist.

Section/Topic	Item	Checklist Item	Location in Manuscript
TITLE	1	Identify the report as a systematic review.	Title
ABSTRACT	2	See the PRISMA 2020 for Abstracts checklist.	Abstract
INTRODUCTION	3	Describe the rationale for the review in the context of existing knowledge.	Introduction
INTRODUCTION	4	Provide an explicit statement of the objective(s) or question(s) the review addresses.	Introduction
METHODS	5	Specify the inclusion and exclusion criteria for the review and how studies were grouped for the syntheses.	Methods—Inclusion Criteria
METHODS	6	Specify all databases, registers, websites, organisations, reference lists and other sources searched or consulted to identify studies. Specify the date when each source was last searched or consulted.	Methods—Search Strategy
METHODS	7	Present the full search strategies for all databases, registers and websites, including any filters and limits used.	Appendix A—Table A1
METHODS	8	Specify the methods used to decide whether a study met the inclusion criteria of the review.	Methods—Inclusion Criteria
METHODS	9	Specify the methods used to collect data from reports.	Methods—Data Extraction
METHODS	10	List and define all outcomes for which data were sought. Describe methods of handling and summarizing the data.	Methods—Outcome Definitions
METHODS	11	Specify the methods used to assess risk of bias in the included studies.	Methods—Risk-of-Bias Assessment
METHODS	12	Specify for each outcome the effect measure(s) used in the synthesis or presentation of results.	Methods—Statistical Analysis
METHODS	13a	Describe the processes used to decide which studies were eligible for each synthesis.	Methods—Subgroup Analysis
METHODS	13b	Describe any methods required to prepare the data for presentation or synthesis.	Methods—Statistical Analysis
METHODS	13c	Describe any methods used to tabulate or visually display results.	Figures and Tables
METHODS	13d	Describe any methods used to synthesize results and provide a rationale for the choice(s).	Methods—Statistical Analysis
METHODS	13e	Describe any methods used to explore possible causes of heterogeneity among study results.	Subgroup Analyses
METHODS	13f	Describe any sensitivity analyses conducted.	Subgroup Analyses
METHODS	14	Describe any methods used to assess certainty (or confidence) in the body of evidence.	Methods—Risk-of-Bias and GRADE
RESULTS	16a	Describe the results of the search and selection process, from the number of records identified to the number of studies included.	Results—Study Selection and Figure 1
RESULTS	16b	Cite studies that might appear to meet the inclusion criteria, but which were excluded, and explain why.	Not applicable (no excluded studies listed)
RESULTS	17	Present the characteristics of included studies.	Table 1
RESULTS	18	Present assessments of risk of bias for each included study.	Table 4 and Results—Risk of Bias
RESULTS	19	Present results of all statistical syntheses conducted.	Results—Symptom Score, Relapse, Safety
RESULTS	20a	For each synthesis, briefly summarize the characteristics and risk of bias among contributing studies.	Results—Subgroup Analysis
RESULTS	20b	Present results of all investigations of possible causes of heterogeneity.	Results—Subgroup Analysis
RESULTS	20c	Present results of all sensitivity analyses conducted to assess the robustness of the synthesized results.	Results—Subgroup Analysis
RESULTS	21	Present assessments of risk of bias due to missing results (arising from reporting biases) for each synthesis assessed.	Not done; too few studies for funnel plot. Stated in Limitations.
RESULTS	22	Present assessments of certainty (or confidence) in the body of evidence for each outcome assessed.	Table 5—GRADE Summary
DISCUSSION	23a	Provide a general interpretation of the results in the context of other evidence.	Discussion
DISCUSSION	23b	Discuss any limitations of the evidence included in the review.	Discussion—Limitations
DISCUSSION	23c	Discuss any limitations of the review processes used.	Discussion—Limitations
DISCUSSION	23d	Discuss implications of the results for practice, policy, and future research.	Discussion—Clinical Implications and Future Research
OTHER	24a	Review registration info.	Methods—‘This review was not registered.’
OTHER	24b	Access to review protocol.	Not applicable. No protocol was prepared.
OTHER	24c	Amendments to protocol.	Not applicable. No protocol prepared.
OTHER	25	Sources of support.	Funding statement
OTHER	26	Competing interests.	Conflicts of Interest section
OTHER	27	Data/material availability.	Data Availability Statement
